# Comparative cost analysis of different prosthetic rehabilitations for the edentulous maxilla: early results from a randomized clinical pilot study

**DOI:** 10.1038/s41405-022-00100-0

**Published:** 2022-03-22

**Authors:** Peyman Ghiasi, Sofia Petrén, Bruno Chrcanovic, Christel Larsson

**Affiliations:** 1 Department of Prosthodontics, Faculty of Odontology, Malmö University, Malmö, Sweden; 2 Department of Orthodontics, Faculty of Odontology, Malmö University, Malmö, Sweden

**Keywords:** Removable prosthodontics, Fixed prosthodontics

## Abstract

**Objectives/aim:**

To analyze and compare costs of different prosthetic rehabilitations for the edentulous maxilla.

**Materials and methods:**

Patients with edentulous maxillae were rehabilitated with either of three implant-supported prosthetic protocols; removable overdenture supported by 2 implants (ISOD 2), fixed dental prostheses supported by 4 (ISFAFDP 4) or 6 (ISFAFDP 6) implants. Cost of treatment and costs during follow-up were registered and compared.

**Results:**

Twenty-four patients were included: six patients received ISOD 2 treatment, eight patients received ISFADP 4 treatment and ten patients received ISFADP 6 treatment. Initial costs for ISFAFDP 6 were higher than costs for ISFAFDP 4 and ISOD 2, but there were no differences in cost for maintenance i.e., the ISOD treatment remained the least costly treatment alternative after 1-year follow-up.

**Discussion:**

The lack of difference in cost for maintenance and repair over the first year suggests that implant-supported overdentures will remain the least costly treatment option for the edentulous maxilla, at least in a short-term perspective.

**Conclusions:**

Removable maxillary overdentures supported by 2 implants may be a valid low cost treatment option.

## Introduction

Edentulism, the loss of all natural teeth, is a debilitating and irreversible condition. While there is a downward trend in edentulism in several countries, it is region-specific, and need for rehabilitation of edentulous patients is likely to remain relevant for the foreseeable future [1]. Edentulism is thus still an important public health issue globally, associated with considerable disability [2, 3]. For the edentulous patient, there are basically three rehabilitation strategies: removable complete denture (RCD), implant-supported overdenture (ISOD), or implant-supported full-arch fixed dental prosthesis (ISFAFDP).

RCD provides the least expensive treatment among these three rehabilitation options, but there is sometimes a considerable dissatisfaction among patients with RCDs, essentially related to suboptimal retention, adaptation difficulties and ensuing sense of insecurity [1, 4]. The ISFAFDP option, which provides a fixed restoration, is a well-documented evidence-based treatment, also resulting in high patient satisfaction [5, 6]. This treatment, however, requires installation of several implants, and more complex manufacturing procedures, resulting in a higher initial cost. The remaining treatment option, ISOD, represents a beneficial option in order to improve retention and stability of a prosthesis with fewer implants and at a reduced initial cost compared to ISFAFDPs [7].

Although a well-established approach for the oral rehabilitation of edentulous patients, ISODs are not without drawbacks. ISODs have been suggested to need constant maintenance follow-ups to address technical complications [8]. In the long run, these incremental maintenance expenses may lead to high total cost of treatment. If the need for maintenance and repair is substantial, an initially less expensive treatment may end up more costly than an initially expensive treatment if there are considerable differences in need and cost of repair. The initial low-cost alternative, the ISOD treatment, could thus become more costly in a longer perspective than the high cost at delivery alternative, the ISFAFDP. However, technical complications also occur among ISFAFDPs [9]. To our knowledge, there is a lack of studies analysing costs of different treatment options for the edentulous maxilla taking both cost at delivery and cost of maintenance and repair into consideration.

The dental profession needs data on costs for different treatments to establish consensus for treatment selection [10, 11]. The intervention that incurs the lowest cost is the one that would be most rational to implement if the goal is to minimize cost [10]. In this respect, economic evaluations have become an integral component of health services. The main reason is that resources within the health sector (personnel, time, facilities, equipment, and knowledge) are limited [10]. Failure to analyze economic aspects of dental health service evaluations may result in either unsustainable overexpenditure, withdrawal, or reduction of services or resources in other areas of healthcare [11]. In allocating resources, including dental care, health service purchasers need to take into account not only evidence of clinical effectiveness of treatment procedures but also relative costs, i.e., “value for money” [12].

In summary, dental implants can improve the retention and stability of dentures, although the treatment cost increases with the number of implants. The initial cost of an ISOD will generally be lower in comparison to ISFAFDP due to the use of fewer implants. The design of an ISOD can, however, vary from full palatal coverage to horse-shoe milled bar fixed-removable hybrid design, where only the former is used with significantly fewer implants compared to the ISFAFDP. Whether an ISOD is Less costly in comparison to ISFAFDP is not entirely clear when several parameters are considered. The aim of the present randomized clinical trial was to analyze the costs of rehabilitation of edentulous patients provided with either ISOD or ISFAFDP treatment from the perspective of both the patient and dentist, and to some extent the overall dental health care system. More specifically, we aimed to evaluate whether a removable overdenture with initial low cost at delivery would remain the lower cost alternative also after 1-year follow-up taking the cost of maintenance and repair into consideration.

The hypothesis was that initial differences in cost at delivery between ISOD and ISFAFD will be reduced by higher need for maintenance in the ISOD group.

### Significance

Knowledge of outcome including comparative cost analysis of different treatment options is of key importance to provide guidance for clinical decisions for dentists and patients alike.

## Materials and methods

The study protocol was approved by the Regional Ethical Review Board in Lund, Sweden (Dnr 2015/751).

### Patients

Between January 2015 and December 2020, 24 patients who met the inclusion criteria were recruited. The study subjects consisted of patients with edentulous maxillae and rehabilitated at the Department of Prosthodontics, Faculty of Odontology, Malmö University, Sweden with either of three prosthetic protocols, namely removable ISOD with full palatal coverage supported by 2 implants, ISFAFDP supported by 4 (ISFAFDP 4) or 6 implants (ISFAFDP 6), in a prospective randomized clinical trial. Before the treatment started, the patients were provided with verbal and written information about the trial and gave their informed consent to participate.

### Inclusion and exclusion criteria

The inclusion criteria were the following: (1) patients of at least 18 years of age, (2) edentulous maxilla, (3) patients encountering problems with the existing dentures and in need or desire for dental implant treatment, (4) patients of good general health condition, without local or systemic contraindications for oral surgery, (5) patients having any ridge resorption pattern in the anterior maxilla [13] provided that implants could be placed with primary stability mostly embedded in autologous bone, and (6) patients willing to participate and have signed an informed consent.

The exclusion criteria consisted of (1) patients with clinical signs of severe oral functional disorders, (2) patients with systemic diseases/conditions jeopardizing successful implant therapy, (3) patients with disorders in the area of planned implant placement, such as chronic bone diseases, present or previous tumors or irradiation, and (4) patients lacking compliance with the study protocol.

### Randomization

Allocation of patients to either test group, ISOD 2 implants or ISFAFDP 4 implants, was randomized at the stage of abutment connection surgery. A dental assistant drew a lot, i.e., an envelope containing a note with either “ISOD 2” or “ISFAFDP 4” written, successively for each patient. ISFAFDP 6 implants (control) were not randomized. This group consisted of patients who were not able to be included in the study due to insufficient bone quality to receive prostheses supported by less than six implants.

### Implant surgery and prosthetic treatment

Implants (Deep Conical, Southern Implants, Irene, South Africa) were placed by one experienced oral and maxillofacial surgeon. Based on the group, the surgeon installed four implants (ISOD and ISFAFDP 4), preferably in the canine area and second premolar or six implants (ISFAFDP 6) in the edentulous maxilla of the patient. In the ISOD group, two implants were to be used as support and two implants left resting. The two dormant implants thus acted as a reserve in case the outcome showed that a two-implant anchorage was not sufficient, or the patient for some reason would rather have an ISFAFDP at the end of the study, or if one of the two active implants would be lost during healing or at a later stage.

The abutment connection was performed 3–4 months after implant placement. After a two-week period of mucosal healing, an impression was made. The ISODs were designed without metal framework and connected to two Locator abutments (OT Equator, Rhein83 Srl, Bologna, Italy). The ISFAFDP were made of milled titanium frameworks with acrylic teeth and screw-retained to abutments (MC-DC3, Southern Implants, Irene, South Africa). All prosthetic treatments were performed by the same operator, a specialist in prosthodontics (PG).

### Follow-up

Post-delivery check-ups were performed within a week after prosthesis delivery. Patients in the two test groups were thereafter called back for 6- and 12-month follow-ups, control patients were called back for a 12-month follow-up. Examinations included clinical and radiological evaluation of implant and prosthesis stability and presence or not of any technical (component or material wear or fracture) or biological complications (peri-implant soft tissue bleeding and/or pocket depth) as well as any patient complaints (esthetic or functional).

### Cost analysis

Dental health care costs vary widely and are influenced by different subsidy systems [14]. In some systems, prosthodontic treatments are fully paid by the patient, in others it is partly or fully subsidized. In the present study, the full cost of treatment without subsidies are analysed to facilitate comparisons nationally and internationally.

The outcome measures to be assessed in the trial were:

Any complications that needed intervention in the form of repair during follow-up, i.e., the total time needed for each visit for maintenance and repair including scheduled and emergency visits and cost of any dental technical laboratory work for repairs.

The comparative cost analysis included two parts:

- post-treatment costs
- post-treatment costs plus initial cost of prosthesis at delivery = total cost of treatment up until 1-year follow-up

Cost of the prostheses at delivery consisted of costs for dentist fees and dental technical laboratory costs including costs for implants and implant components. These costs differ between treatment groups but do not differ between individuals in each respective treatment group. Differences regarding costs of the prostheses at delivery were therefore not compared between groups. Instead, post-treatment costs for maintenance and repair were analyzed and compared. The number of appointments and treatment time (in minutes) for maintenance and repair, were registered for every patient together with any costs for dental technical laboratory work for repairs. In addition, post-treatment costs plus costs of the prostheses at delivery were combined to form “total cost of treatment” after 1 year. This cost was analyzed per treatment group and compared.

All costs were based on 2021 prices and expressed in Euros. Costs in Swedish kronor, SEK, were converted to Euro, EUR, using a web-based currency converter (www.xe.com). Calculations were performed at the time of preparing the manuscript (May 2021). The cost of 1 SEK equaled 0.00987 EUR which was rounded up to the nearest ten and expressed as 10 SEK = 1 Euro. Hourly rates were set at 3000 SEK/hour = 300 Euro/hour i.e., 5 Euro /minute according to the clinics tariff.

### Statistical analysis

The economic costs were calculated using score values and the results were analyzed using One-Way ANOVA and Kruskal-Wallis test. SPSS software (SPSS, Version 27, IBM Co., Chicago, IL, USA) was used to perform the statistical analyses of the data. Numerical variables were described with mean (± standard deviation, SD). A significance level of *p* < 0.05 was used.

## Results

Twenty-four patients were included, seven women and seventeen men, mean age 64.9 and 67.5, respectively. Six patients received ISOD 2 treatment (2 women, mean age 62.5 and 4 men, mean age 63.5). Eight patients received ISFADP 4 treatment (8 men, mean age 67.9). Ten patients received ISFADP 6 (5 women, mean age 65.8 and 5 men, mean age 70.0).

All implants and all restorations were in function at follow-up, i.e. the survival rate was 100%. Initial costs, i.e., cost of prostheses at delivery, for ISFAFDP 6 were higher than costs for ISFAFDP 4 and ISOD 2, due to the higher number of implants and higher cost of materials and fees Table 1.

**Table 1:** Costs of prostheses at delivery (dentist fees and dental technical laboratory costs including costs for implants and implant components).

Treatment	Costs (Euro)
ISOD 2	4519
ISFADP 4	9341
ISFADP 6	11,475

Three complications occurred in the ISOD 2 group, two in the ISFAFDP 4 group, and three in the ISFAFDP 6 group. Table 2 There were no statistically significant differences between groups in post-treatment costs (*p* > 0.05) Table 3.

**Table 2:** Post-treatment events and costs: number of appointments, treatment time (minutes) and costs (Euro) for dental technical laboratory work.

	Complication	Cost of repair	Chair time (minutes)
ISOD 2	Denture fracture	120 + 198	2 × 30
	Change of retentive element × 2	- (chairside repair) 285 × 2	20
	Change of retentive element × 4	- (chairside repair) 285 × 4	20
ISFAFDP 4	Chip off	100 + 259	2 × 50
	Chip off	100 + 259	2 × 50
ISFAFDP 6	Chip off	100 + 259	2 × 50
	Chip off × 2	200 + 259 × 2	4 × 50

**Table 3:** Post-treatment costs per group (Euro).

Groups	*n*	Mean ± SD	(min, max)
ISOD 2	6	1 648 ± 2 405	(0, 6180)
ISFADP 4	8	2 148 ± 3 976	(0, 8590)
ISFADP 6	10	2 577 ± 5 798	(0, 17,180)

Comparison between all three groups together: *p* = 0.745 (Kruskall–Wallis test).

*SD* standard deviation.

There were significant differences between groups regarding total costs, i.e., costs of prostheses at delivery plus post-treatment costs. Total costs for ISFAFDP 6 were significantly higher than total costs for ISFAFDP 4 (*p* < 0.001) and ISOD 2 (*p* < 0.001). Total costs for ISFAFDP 4 were significantly higher than total costs for ISOD 2 (*p* = 0.001) Table 4.

**Table 4:** Total cost (Euro) (Patient’s initial cost + post-treatment).

Groups	*n*	Mean ± SD	(min, max)
ISOD 2	6	46 833 ± 2 406	(45,185, 51,365)
ISFADP 4	8	95 553 ± 3 976	(93,405, 101,995)
ISFADP 6	10	117 322 ± 5 798	(114,745, 131,925)

Comparison between all three groups together: *p* < 0.001 (Kruskall-Wallis test).

*SD* standard deviation.

## Discussion

This study showed that three different treatment options for the edentulous maxilla yielded similar survival rate and similar amount of post-treatment maintenance and repair in the short term of 12 months, with no difference in costs. The initial substantial differences in cost between the fixed and the removable treatment alternatives remained after the 1-year follow-up, thus the hypothesis is rejected.

The fact that total costs differed is unsurprising considering the substantial differences in initial costs of treatment, due to increased number of implants together with increased complexity in design and manufacturing for ISFAFDPs. Previous literature suggests similar findings [15]. Absence of significant differences in costs for maintenance and repair, suggests that total costs may remain significantly different even in a long-term perspective as further minor repair will not offset the considerable differences in initial costs. This suggestion is however dependent on the type of complications that occur. Complications such as minor fractures of material or retentive components are easily adjusted. Most complications that occurred in the present short-term evaluation incurred minor costs. Major fractures of prostheses, and loss of implants on the other hand, may cause vast post-treatment expense if surgical intervention with additional implants and significant adaptation of existing prosthesis, or even a new prosthesis, is needed.

A recent consensus report found high overall prosthesis survival for removable as well as fixed complete implant prostheses, albeit with high rates of technical complications for the fixed restorations [16]. Chipping or fracture of veneering material was the most frequent technical complication for fixed restorations, whereas attachment-related complications were the main issue for removable overdentures. The consensus report found insufficient data to perform a cost-effectiveness analysis but it is likely that the complications that were common in the fixed group are more expensive to adjust as they may require removal of the restoration and assistance of a dental technician for the repair. Other studies have noted that overdentures require constant maintenance regardless of the type of attachments system, but changing parts is time- and cost effective and additional work and expenses are minimal [7, 8]. The most serious complication, implant loss, was however more frequent among removable implant-supported prostheses. These differences in type of maintenance could affect long-term costs. In this perspective, further follow-up of this patient group is relevant.

Other studies have concluded that dental care utilization is related to attitudes towards costs. The cost of treatment is one of the more influential factors and one that is of importance for patients as well as dental professionals and society in general [17]. Patients’ cost of treatment is influenced by insurance systems. Generous subsidies may gear treatment choice towards fixed restorations over removable ones when the insurance system provides substantial subsidies for expensive treatments which could influence patients’ choice of treatment. The full cost of treatment, without subsidies, were the basis of analysis in the present study, to facilitate comparisons nationally and internationally.

The patients in the present study were not affected by costs for maintenance and repair as such work is included in a compulsory national warranty in Sweden. However, in other countries these events would incur a cost. Furthermore, irrespective of subsidiary system and warranties the patient is affected in the form of time spent in the dentist office, potential loss of income and cost of travel.

All costs for maintenance and repair in the present study instead affect the dentist. The additional dental technical laboratory work affects the dentist as a cost that cannot be charged to the patient as repairs during the first years are included in a compulsory warranty to protect the patient. In addition, the time spent becomes an additional cost in the form of loss of income. Time spent on maintenance and repairs also affect the overall provision of dental health care as this time could have been used to tend to other patients in need of dental treatment.

Public health developers are responsible for planning, executing, and evaluating public health programs and must determine the most appropriate programs and policies. To choose between competing alternatives, two aspects of a treatment must be considered; outcome and cost [15, 18]. Cost analysis in prosthetic dentistry allows the comparison of costs across different interventions and provides useful input for the dentist in giving objective and correct information to patients in relation to treatment alternatives. The economic cost of any treatment is perhaps particularly important for the ageing population when income streams may be decreasing or limited [19]. It is in this group that we find the highest prevalence of edentulous patients.

### Limitations

The most significant limitation in the present study is the number of patients and the one-year follow-up that represents only short-term information. However, most prosthesis failure occurs due to loss of implants, where the first year seems to be the most critical period [20]. The study may thus provide relevant results despite being a short-term pilot trial.

Indirect costs such as loss of income, transportation etc. were not included. This was a deliberate decision based on the fact that the majority of patients were retired with similar income from pensions and residing close to the clinic, i.e., it was concluded that indirect costs would not differ between groups and would therefore not be a relevant variable.

## Conclusions

This study found excellent and similar clinical performance for fixed and removable implant-supported restorations with no differences in cost for maintenance and repair in a short-term perspective. The lack of difference in cost for maintenance and repair over the first year suggests that implant-supported overdentures will remain the least costly treatment option for the edentulous maxilla, at least in a short-term perspective.

The results of this study have to be evaluated carefully because of the small number of patients examined and the short observation period.
